# Impact of Food and Drink Administration Vehicles on Paediatric Formulation Performance: Part 1—Effects on Solubility of Poorly Soluble Drugs

**DOI:** 10.1208/s12249-020-01722-z

**Published:** 2020-06-26

**Authors:** J. Martir, T. Flanagan, J. Mann, N. Fotaki

**Affiliations:** 1 Department of Pharmacy and Pharmacology, University of Bath, Claverton Down, Bath, BA2 7AY UK; 2 Oral Product Development, Pharmaceutical Technology & Development, Operations, AstraZeneca, Macclesfield, UK; 3 Currently at UCB Pharma, Chemin du Foriest, B-1420 Braine-l’Alleud, Belgium

**Keywords:** drug manipulation, food, drinks, solubility, physicochemical properties, multivariate analysis, paediatrics

## Abstract

Food and drinks are commonly used to facilitate administration of paediatric medicines to improve palatability and enhance patient compliance. However, the impact of this practice on drug solubility and on oral drug bioavailability is not usually studied. Based on recommended strategies for oral administration of paediatric medicines with food and drink vehicles, the aims of this study were (i) to measure the physicochemical properties of (soft) food and drink vehicles, commonly mixed with paediatric medicines prior to administration, and (ii) to assess the impact of the co-administered vehicles on the solubility of two poorly soluble paediatric drugs. Montelukast (sodium) and mesalazine were selected as the model compounds. Distinct differences were observed between the physicochemical properties (*i.e.* pH, surface tension, osmolality, viscosity and buffer capacity) and macronutrient composition (*i.e.* fat, sugar and protein content) of the different soft foods and drinks, not only among vehicle type but also within vehicles of the same subtype. Solubility studies of the two model compounds in selected drinks and soft foods resulted in considerably different drug solubility values in each vehicle. The solubility of the drugs was significantly affected by the vehicle physicochemical properties and macronutrient composition, with the solubility of montelukast being driven by the pH, fat and protein content of the vehicles and the solubility of mesalazine by vehicle osmolality, viscosity and sugar content. This vehicle-dependent impact on drug solubility could compromise its bioavailability, and ultimately affect the safety and/or efficacy of the drug and should be taken into consideration during paediatric product development.

## INTRODUCTION

Paediatric formulation development has been marked by new regulations, additional funding opportunities and research initiatives in both the USA and Europe. Nevertheless, development of acceptable, age-appropriate dosage forms, whilst maintaining safety and efficacy and ensuring compliance, remains a challenge due to the unique requirements and limitations of this heterogeneous population (1,2).

Healthcare professionals, parents and carers still face the need to manipulate medicines designed for adults in order to adapt dosage forms to give smaller doses, improve palatability and enhance compliance amongst paediatric patients (3). This manipulation can range from simple (*e.g.* tablet splitting) to more complex methods (*e.g.* tablet crushing for suspension preparation). A common practice is to mix medications with food or drink vehicles to mask the unsatisfactory palatability of a formulation, in cases that it cannot be improved through dosage form design, and/or to enhance acceptability through swallowing facilitation or texture improvement (4–6).

When this practice is intended, appropriate compatibility studies should be conducted in order to assess compatibility issues and evaluate the possible impact on drug bioavailability (7). Clear instructions on the type of vehicles appropriate for mixing with the medicine should be provided in the patient information leaflet (PIL), summary of product characteristics (SmPCs) and product labelling (7,8). Similarly, appropriate warnings should be provided in cases that such practice is unsuitable, or has not yet been studied, with any mixing outside the recommendations being of the responsibility of the health care professional, patient, parent or carer (8).

In practice, the scientific rationale for co-administering a particular type of vehicle is often not evident (4). Most of the vehicles that appear in the paediatric dosing recommendations of SmPCs and PILs are chosen based on their taste and texture being child-friendly, and there is no general rule on how to administer oral medicines to the paediatric population in a safe and effective way (5,9). Moreover, because of cultural differences in flavour preferences and accessibility of foods around the globe, different vehicles may be used to achieve adequate patient acceptability.

Carers often overlook the recommendations given in SmPCs and PILs, and consequently the clinical implications of this practice of medicine co-administration on drug behaviour and oral drug bioavailability are often not considered. Previous studies have shown that different foods or drinks can have dissimilar effects on the paediatric medicine *in vivo* performance due to their physicochemical properties. For example, the pH of pudding (pH 5.6) damaged the enteric coating of duloxetine pellets and affected its absorption compared to when the pellets were mixed with applesauce or apple juice (10); and the viscosity of applesauce affected dissolution from warfarin crushed tablets in comparison to when these were mixed with orange juice (11).

In an effort to provide guidance on medicine co-administration, the FDA has recently launched a draft guidance entitled ‘Use of liquids and/or soft foods as vehicles for drug administration: general considerations for selection and in vitro methods for product quality assessments’ (7). It is stated that the best vehicles to use for this clinical practice are those with relatively small fluctuations in their macronutrient composition and physicochemical characteristics, such as vehicle viscosity and pH. Furthermore, vehicle candidates should be screened concerning their interaction with drug/formulation and their adequacy to the target age group. This could guide an appropriate use of the vehicle and avoid possible clinical implications (7).

Knowledge of the composition and properties of the food and drinks will aid understanding of their *in vivo* impact on the drug product behaviour. Oral drug performance is influenced by drug bioavailability, which in turn is largely dependent on the drug available in the GI tract to undergo absorption (12). For poorly soluble compounds, oral drug absorption will be limited by drug solubility. Therefore, it is important to understand the impact of medicine co-administration with food and drinks on the behaviour of different drugs. The solubility of a drug serves as a surrogate indicator of oral biopharmaceutical performance and is one of the two factors that are used in the Biopharmaceutics Classification System (BCS) (13). It depends on the physicochemical properties of the drug and the composition of the dissolution medium the drug is exposed to; thus, it can be affected by the co-administered vehicle. To our knowledge, little attention has been devoted to characterising soft foods and drinks commonly used in practice as well as identifying the impact of these properties on drug solubility.

The aims of the present study were (i) to measure the physicochemical properties of a number of food and drink vehicles that are commonly co-administered with paediatric medicines and (ii) to investigate the impact of the co-administered vehicle on the solubility of two poorly soluble paediatric drugs.

The characteristics of the model drugs to study were restricted to include a poorly soluble compound, with pH-dependent solubility, documented usage in both children and adults and recommended to be mixed with food or drink vehicles to facilitate administration in the paediatric population. Based on these criteria, montelukast (sodium) and mesalazine were selected.

Montelukast is a BCS class II compound with low aqueous solubility (0.2–0.5 μg/mL at 25°C (14)), two pKas—2.7 (strongest basic) and 5.8 (strongest acidic) (15) and a clogP of 8.79 (16). Instructions about the use of a paediatric montelukast formulation (Singulair® granules) report that the granules can be mixed with one teaspoonful of soft food (cold or at room temperature) (17).

Mesalazine has been classified as a BCS class IV drug, having an aqueous solubility of 0.84 mg/mL at 25°C and a clogP of 0.98 (18). It is a zwitterion having a carboxyl group (–COOH) with a pKa value of 2.3 and an amino group [(NH3^+^)−] with a pKa of 5.69 (19). A commercially available mesalazine formulation (Pentasa® granules) is recommended to be mixed with juice or water to facilitate administration (17).

## MATERIALS AND METHODS

### Materials

Ammonium acetate [high-performance liquid chromatography (HPLC) grade], 37% hydrochloric acid, sodium hydroxide, sodium chloride, sodium acetate trihydrate, glacial acetic acid, sodium phosphate anhydrous, acetonitrile (HPLC grade) and methanol (HPLC grade) were purchased from Fisher Scientific(UK). Trifluoroacetic acid [TFA] (HPLC grade), montelukast sodium and mesalazine were obtained from Sigma-Aldrich Company Ltd. (UK). Water was ultra-pure (Milli-Q) laboratory grade.

Polytetrafluoroethylene [PTFE] filters (0.45 μm), RC filter papers (0.45 μm) (Whatman®, UK) and regenerated cellulose [RC] membrane filters (0.45 μm) (Cronus®, UK) were used.

Based on the recommendations gathered from the UK National (BNF-C (17)) and Hospital (20) formularies and taking into consideration the availability in a clinical setting, 26 different vehicles were selected and characterised. The origin, description, nutritional factors and manufacturer’s preparation instructions of the vehicles studied are described in Table I. Honey, jam, Coca-Cola as well as all squashes, milks, yoghurts, Bramley’s applesauce (Bramley applesauce Colman’s of Norwich, UK) and juices were purchased from The Co-Operative (UK). Three infant formulas were used in the study: First Infant Milk (cow’s milk-based formula) and Infasoy (soya-based formula) (Cow & Gate, UK) and Wysoy (soya-based formula) (SMA-Nestlé, UK). Vehicles with considerably different compositions available in different countries were also analysed. Mott’s natural applesauce (Mott’s LLP, USA) and Bauck Hof applesauce (Bauck Hof Apfelmark, Germany) were purchased from Amazon (UK) and were specifically chosen due to their different composition and region of origin.

**Table I:** Identification, Origin, Nutritional Facts and Instructions for Preparation (When Applicable) of the Vehicles Studied. The Vehicles, Divided in Two Categories—Soft Foods and Drinks—Were Further Categorised into 9 Subgroups

Vehicles		Brand/country	Energy (kJ/kcal)	Nutrition (per 100 mL or g)			Instructions for preparation
				Protein content (g)	Fat content (g)	Sugar content (g)	
Formula	Soya Wysoy	SMA (UK)	281/67	1.8	3.6	2.5	1 scoop of product for each 30 mL of water
	First milk	Cow & Gate First infant milk from newborn (UK)	257/60	1.3	3.4	7.3	
	Infasoy	Cow & Gate Infasoy (UK)	275/66	1.6	3.5	1.0	
Milk	Whole Fresh	The Co-Op (UK)	270/65	3.2	3.6	4.7	N/A
	Skimmed Fresh	The Co-Op (UK)	150/35	3.4	0.1	5.0	N/A
	Whole U.H.T	The Co-Op (UK)	280/70	3.3	4.0	4.7	N/A
	Soya	Alpro Soya (Belgium)	167/40	3.0	1.8	2.8	N/A
	Lactose free (*Semi-skimmed*)	Lactofree, Arla (Denmark)	160/40	3.6	1.5	3.0	N/A
Yoghurt	Plain	Yeo Valley (UK)	344/82	4.6	4.2	6.5	N/A
	Soya (*Alpro soya with yoghurt cultures*)	Alpro (UK)	212/50	4.0	2.3	2.1	N/A
	Lemon curd	Yeo Valley (UK)	536/127	4.7	4.4	16.9	N/A
	Double flavour (*Munch Bunch double Up Strawberry and Banana Yoghurt*)	Nestlé (Switzerland)	432/102	6.1	2.7	12.5	N/A
	Greek (*Greek recipe strained yoghurt total 0%)*	Fage (Greece)	243/57	10.3	0.0	4.0	N/A
	Liquid Strawberry (*Actimel for Kids Strawberry*)	Danone (France)	312/74	3.3	1.3	11.2	N/A
	Strawberry Fromage (*Strawberry Fromage Frais*)	Yoplait (USA)	399/95	5.3	2.3	9.9	N/A
Juice	Apple (*clear*)	The Co-Op (UK)	190/45	< 0.5	< 0.5	9.2	N/A
	Orange (*smooth*)	The Co-Op (UK)	180/42	0.5	< 0.5	9.2	N/A
Coca-Cola	Coca-Cola (*Original*)	The Coca-Cola company (UK)	180/42	0.0	0.0	10.6	N/A
Squash	Blackcurrant Ribena	Lucozade Ribena Suntory Ltd. (UK)	183/43	0.0	0.0	10.0	50 mL of product diluted in 250 mL of water
	Blackcurrant Co-Op	The Co-Op (UK)	90/20	0.5	0.0	3.5	
	Orange	The Co-Op (UK)	60/15	0.2	0.0	1.7	25 mL of product diluted in 250 mL of water
Applesauce	Bramley’s UK	Bramley applesauce Colman’s of Norwich (UK)	481/111	< 0.5	< 0.5	20.0	N/A
	Mott’s Natural US	Mott’s LLP (USA)	171/41	0.0	0.0	4.7	N/A
	Bauck Hof DE	Bauck Hof Apfelmark (Germany)	204/48	< 0.5	0.3	8.7	N/A
Honey	*Clear*	The Co-Op (UK)	270/65	0.1	0.2	80.8	N/A
Jam	Strawberry	The Co-Op (UK)	1064/251	< 0.5	< 0.5	49.0	N/A

*N/A* not applicable

### Methods

#### Preparation of Vehicles and Media

USP-simulated gastric fluid sine pepsin (SGF*sp*) pH 1.2, acetate buffer pH 4.5 and phosphate buffer pH 6.8 were prepared following the USP 27 (21).

Prior to all analysis, squashes and formulas were prepared as per manufacturer’s instructions (Table I) and Coca-Cola was degassed. The dilution of the prepared squashes was not the same (blackcurrant: diluted 1/5 with water; orange: diluted 1/10 with water; Table I). To evaluate if these differences in dilution had an effect on the physicochemical characteristics measured, confirmatory studies were performed with orange squash diluted on a 1/5 (concentrated squash/water) ratio. Results showed that the dilution of the squashes did not have a significant effect on the differences observed in the physicochemical properties measured (data not shown).

#### Physicochemical Characterisation of the Vehicles

Physicochemical characterisation of all vehicles included measurement of pH, buffer capacity, osmolality, surface tension and viscosity. All experiments were run in triplicate and results are expressed as mean values ± standard deviation (SD).

##### pH

The pH of each vehicle was measured, at room temperature, using a pH meter (Mettler Toledo S220 Seven Compact pH/Ion meter, Schwerzenbach, Switzerland). pH measurements took place immediately after opening the soft food/drink container or after vehicle preparation (in the case of the formulas, squashes and Coca-Cola), and agitating the vehicle with a spatula for 5 s.

##### Buffer Capacity

Buffer capacity was quantified by dropwise addition of 0.1 N sodium hydroxide or 0.1 N hydrochloric acid, measuring the volume required to change the pH by one unit, under constant agitation. Buffer capacity was then calculated using the following equation (Eq. 1) (22):

1 $$ \frac{\mathrm{dB}}{\mathrm{dpH}}=\frac{\ \left(\begin{array}{c}\mathrm{cc}.\mathrm{acid}\ \mathrm{or}\ \mathrm{base}\ \mathrm{added}\\ {}\mathrm{to}\ \mathrm{cause}\ \mathrm{pH}\ \mathrm{change}\end{array}\right)\left(\begin{array}{c}\mathrm{normality}\ \mathrm{factor}\\ {}\ \mathrm{of}\ \mathrm{acid}\ \mathrm{or}\ \mathrm{base}\end{array}\right)}{\left(\begin{array}{c}\mathrm{average}\ \mathrm{volume}\ \mathrm{of}\ \mathrm{sample}\\ {}\ \mathrm{over}\ \mathrm{range}\ \mathrm{involved}\end{array}\right)\left(\Delta  \mathrm{pH}\right)} $$

where $\frac{\mathrm{dB}}{\mathrm{dpH}}$ is the buffer capacity, *cc*. is the concentration of acid or base added and ∆pH is the pH change produced.

##### Osmolality

Osmolality was measured *via* freezing-point depression method by a micro-osmometer (Advanced Instruments Inc. micro-osmometer Model 3300, Norwood, MA). Twenty microlitres of sample was placed into the sampler, which was then inserted into the instrument’s operating cradle, and subsequently lowered to the freezing chamber; this initiated the process of super cooling the sample. Following a solenoid-induced pulse and subsequent sample freezing, the liberated heat of fusion was related by a microprocessor to the sample’s freezing point and osmolality was shown on a digital display (23).

The osmolality values of Bramley’s applesauce (UK), honey and jam were quantified based on a set of appropriate dilutions of the vehicles in demineralised water (% (*w*/*w*) vehicle/water). Concentration of vehicle (% (*w*/*w*)) and the osmolality value measured were correlated, and the osmolality value of the undiluted vehicle (*i.e.* 100% (*w*/*w*) vehicle/water) was calculated from the linear regression.

##### Surface Tension

Surface tension was measured with the du Nouy ring method (24), using a ring tensiometer (Sigma 700 Force tensiometer, Attension, UK). Ten millilitres of sample was placed into a glass vessel (Ø = 46 mm) and temperature was set to 25°C. The ring was submerged below the interface of the sample by moving the stage where the vessel was placed. After immersion, the stage was gradually decreased, and the ring pulled up the meniscus of the sample. The force required to raise the ring from the meniscus was measured and used to determine the surface tension.

##### Viscosity

Viscosity of the vehicles was determined using a rheometer (Bohlin Rheometer C-VOR, Malvern instruments, UK) fitted with a cone and plate geometry (4° cone angle, 40 mm diameter). Samples were added to the plate of the rheometer and analysis was carried out at 25°C. Viscosity was measured at increasing shear stress (in the range of 0.1 to 4 Pa) for the drinks (modification of (25)) and increasing shear rate (from 0.1 to 85 s^−1^) for the soft foods (modification of (26)), with 10 s delay time and 10 s integration time at each shear. While the rheological curves for each sample were measured, for simplicity, the viscosity value used for statistical analysis was η50 (*i.e.* the measurement at a shear rate of 50 s^−1^), which is the shear rate most often associated with swallowing (11).

#### Chromatographic Conditions

Drug quantification was performed with HPLC with ultraviolet (UV) detection. Samples were analysed with an Agilent HPLC system 1100 series (montelukast) and 1200 series (mesalazine) (Agilent Technologies, USA). The HPLC method used for the analysis of montelukast is a modification of a published method (27). A reversed-phase (RP) J.T. Baker Octadecyl-C_18_ column (250 mm × 4.6 mm, 5 μm particle size) was used. The mobile phase was composed of ammonium acetate buffer pH 5.6 and methanol (solvents A and B, respectively) delivered at a flow rate of 1 mL min^−1^. The selected gradient started with 10% of solvent B, which was increased linearly to 50% over 2 min, and linearly to 90% between 2 and 4 min; at 11.30 min, the initial conditions of analysis were re-established. Injection volume was 100 μL. Analysis was performed at 20°C and the detection wavelength was 284 nm. The HPLC method used for mesalazine analysis is a modification of a published method (18). A RP Agilent Eclipse XBD-C_18_ column (250 mm × 4.6 mm, 5 μm particle size) was used. The mobile phase was composed of methanol and 0.05% TFA-Water (5:95) delivered at a flow rate of 1 mL min^−1^. Injection volume was 20 μL. Analysis was performed at 40°C and the detection wavelength was 304 nm.

#### Solubility Studies

Solubility studies of montelukast and mesalazine were performed in 16 food and drink vehicles; these included formula (first milk), milk (whole U.H.T), yoghurts (plain flavour, lemon curd and Greek), juices (apple and orange), Coca-Cola, squashes (blackcurrant Ribena®, orange and blackcurrant Co-Op®), honey, jam and applesauces (Mott’s natural applesauce US, Bramley’s applesauce UK and Bauck Hof applesauce DE). Solubility studies of the two compounds were also performed in USP SGF*sp* pH 1.2, acetate buffer pH 4.5 and phosphate buffer pH 6.8 to compare between drug solubility in these media and in different food and drinks of corresponding pH and investigate the effect of media pH on the solubility of the compounds.

An excess amount of drug was added to 1.5 g of foods and 1.5 mL of buffers/drinks, in centrifuge tubes and stirred with a spatula for 30 s. A pilot study was performed with different amounts of drug added to selected vehicles (*i.e.* formula, blackcurrant squash Ribena, Greek yoghurt, honey, applesauce DE and jam) to assess the impact of drug excess amount on drug solubility; drug solubility results were not affected by the amount of drug used in the study (data not shown). Capped tubes were placed in a shaking water bath (37°C) (Grant SS40-2, Grant Instruments, UK), under constant shaking rate of 200 strokes/min, and protected from light to avoid photodegradation (28,29). Samples were collected at 4 h and 24 h. Undissolved drug was removed by centrifugation (Eppendorf Heraeus Fresco 17 centrifuge, Thermo Electron LED GmbH, Germany) at 8000 rpm for 15 min, at 4°C. A total of 1000 μL of acetonitrile (montelukast) or 500 μL of 10% (*v*/*v*) TFA/water (mesalazine) were then added to 500 μL (or mg) of the centrifuged sample. The mixture was vortexed for 1 min and centrifuged. The supernatant was then filtered through a RC (montelukast) or PTFE (mesalazine) filter (0.45 μm), placed into amber HPLC vials and analysed. Honey and jam montelukast samples were diluted (dilution 1:2) with a solution of acetonitrile/water (1:1) prior to the treatment step.

Centrifugation technique was confirmed and validated as an efficient separation method of undissolved drug, after three investigational studies were performed in selected vehicles (*i.e.* whole milk, orange juice, applesauce UK, plain yoghurt, Greek yoghurt). These were (i) filtration of saturated drink samples and comparison of drug solubility results with those obtained with centrifugation technique, (ii) filtration of the supernatant after centrifugation of saturated samples and comparison with drug solubility results obtained when the supernatant was not filtered and (iii) different centrifugation conditions (speed and time) and sequential centrifugations were tested and compared with drug solubility results obtained with original centrifugation conditions (data not shown).

All experiments were performed in triplicate. Quantification of the concentration of drug in samples was performed based on calibration curves. Fresh calibration curves (concentration range: 0.2–100 μg/mL (montelukast) and 5–200 μg/mL (mesalazine)) were prepared in the corresponding media (buffer or vehicle), by appropriate dilution of a 1000 μg/mL stock solution of the analytical standard in methanol (montelukast) or 0.05% TFA/water (mesalazine); the same treatment process was applied as described for the samples.

#### Data Analysis

Vehicle characterisation data was analysed with one-way ANOVA using Statgraphics Centurion XVII software (Statpoint Technologies Inc., USA). *Post hoc* analysis was performed using Tukey honest significant difference (HSD) test, in order to perform pairwise multiple comparison of between vehicles of the same subtype (*p* < 0.05 noting statistical significance).

Drug solubility results obtained in all studied vehicles were correlated to the physicochemical properties (pH, buffer capacity, surface tension, viscosity, osmolality) and macronutrient composition (percentage of fat, sugars and proteins) of the vehicles and selected interactions by partial least square regression (PLS-R) analysis using XLSTAT Software (Microsoft®). The interactions selected as independent variables were (i. interactions of vehicle pH with all other independent factors (physicochemical properties and macronutrient composition of the vehicles), chosen due to the difference between drug solubility in simple buffers and in vehicles of corresponding pH, and (ii) interactions between vehicle viscosity and macronutrient composition, chosen due to the differences observed for drug solubility in the different soft foods.

PLS-R analysis is a statistical method which relates multivariate descriptor sets to different response sets (30). Four PLS-R models were constructed: one for the solubility of each drug at each time-point studied (4 and 24 h). The quality of the models produced was assessed by R^2^ and Q^2^, which measure the fraction of the total variation of the response explained by the model and the fraction of the total variation of the response that can be predicted by the model, respectively. *Q*^2^ and *R*^2^ values above 0.5 and 0.8 refer to a model with good fit and prediction power, respectively (31). The statistical analysis generates components, based on the independent variables set to explain the response. These components are built iteratively so as to better explain the variability of the dependent variable (response), and their number is lower than the initial variable input into the model (30). The PLS-R models were built and evaluated based on full cross-validation (leave-one-out procedure). The number of principal components for each model was selected based on the optimum *Q*^2^ value. The variable influence on projection (VIP) function, which describes the importance of the factors for the response cumulatively, was used to identify which factors were most relevant for explaining drug solubility (with VIP > 1 noting statistical significance) (30). The standardised coefficients were used to indicate the relative impact (positive or negative) of each factor or interaction on drug solubility.

## RESULTS AND DISCUSSION

### Physicochemical Characterisation of the Food and Drink Vehicles

**Fig. 1:**
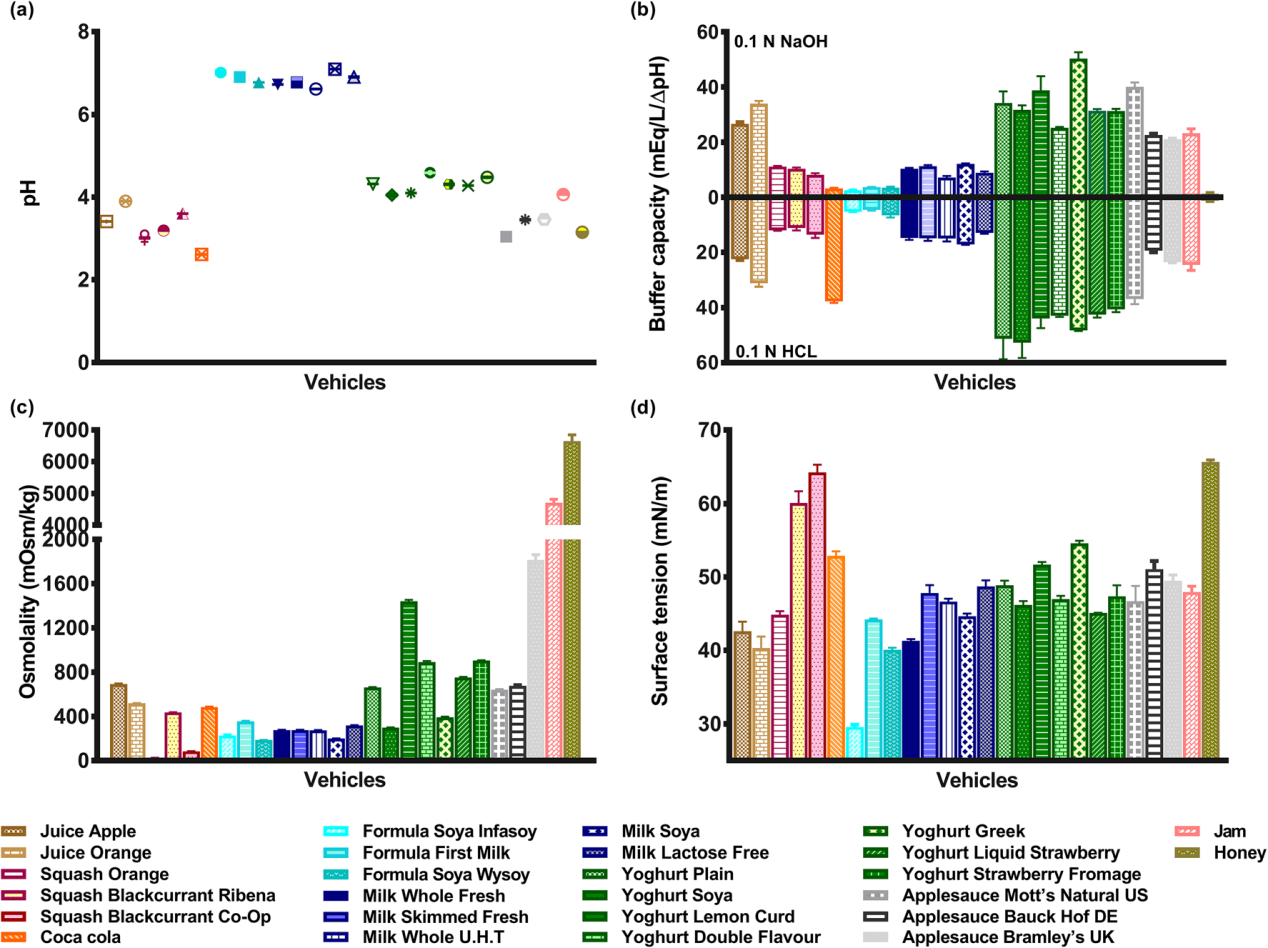
Physicochemical properties of 26 vehicles used for co-administration of drugs: **a** pH. **b** Buffer capacity, per addition of NaOH (upper part) or HCl (lower part). **c** Osmolality. **d** Surface tension [each set of colours represents a subtype of vehicles]

**Fig. 2:**
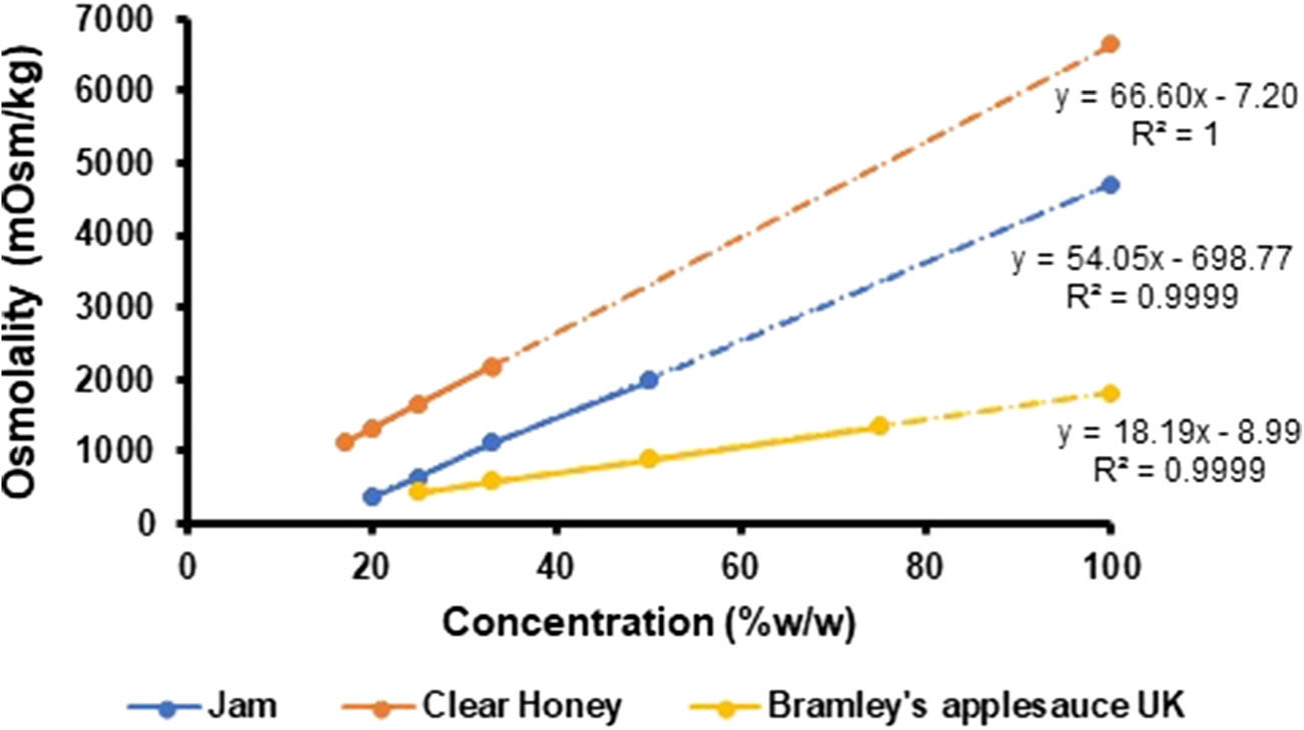
Linear regression of the osmolality values measured for the different mixtures vehicle (jam, honey and Bramley’s applesauce US)/water (% (*w*/*w*)), used to extrapolate the osmolality value of the undiluted vehicles (dashed lines)

**Fig. 3:**
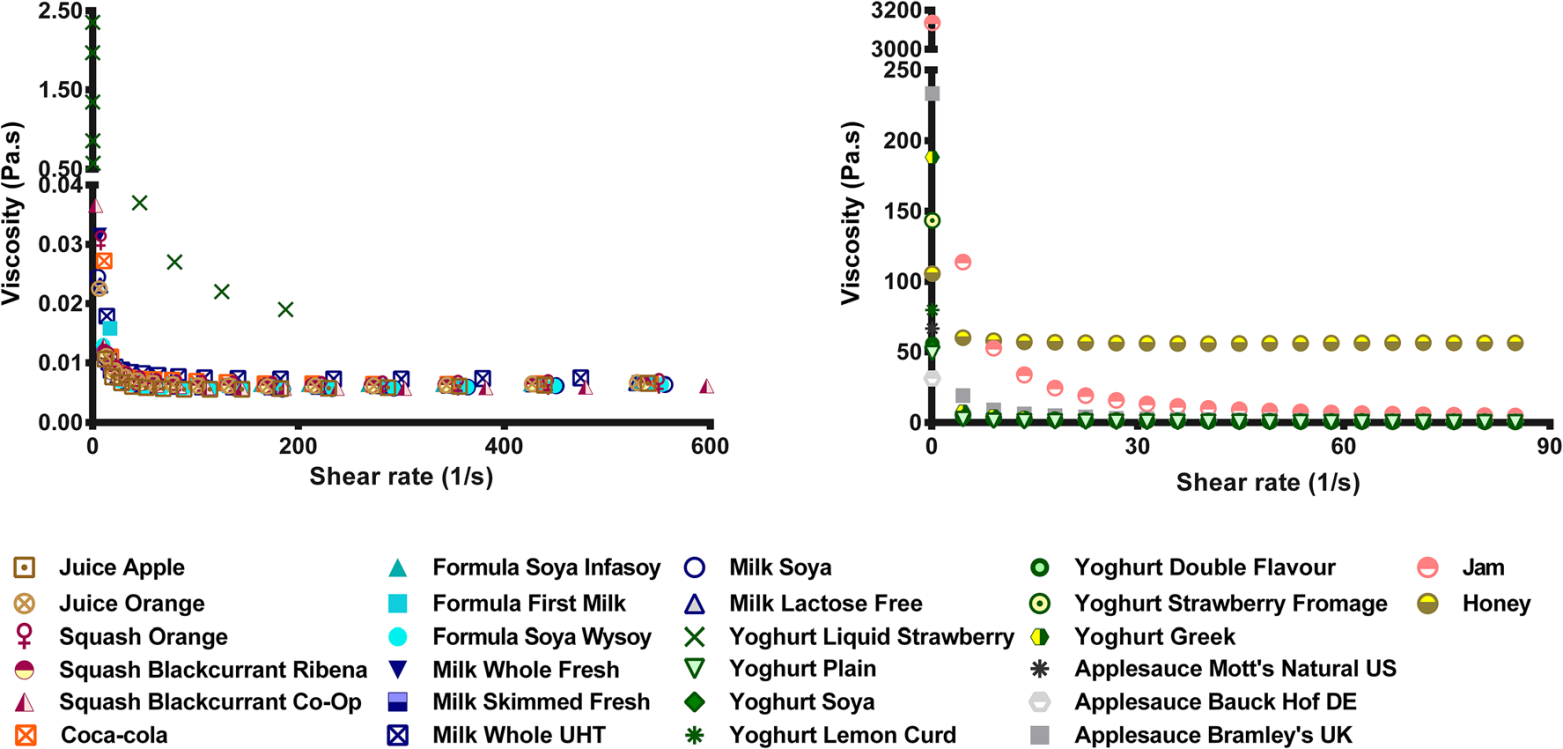
Viscosity of the drinks and soft foods measured at increasing shear stress (0.1 to 4 Pa) for the drinks (left panel) and increasing shear rate (0.1 to 85 s^−1^) for the foods (right panel)

Results from the physicochemical characterisation (pH, buffer capacity, osmolality, surface tension and viscosity) of the 26 selected vehicles are shown in Figs. 1, 2 and 3.

#### pH

pH values measured were in the range of 3 to 4 for ‘fruity’ vehicles (*i.e.* squashes, juices, Coca-Cola, applesauce), pH range 4 to 4.5 for ‘milky’ soft foods (*i.e.* yoghurts) and pH range 6 to 7 for ‘milky’ drinks (*i.e.* milk, formulas) (Fig. 1a).

As observed in the results, the pH of food and drinks of the same subtype is usually controlled within a specific range of pH, mostly due to their composition (32). For example, the pH of yoghurts in the range of 4 to 4.5 can be explained by the use of bacteria (normally, *lactobacillus acidophillus*) in their manufacturing process to convert milk sugar/lactose into lactic acid, which ultimately increases the acidity of the product (33). The pH of applesauces, orange and apple juice will be close to the pH of the corresponding fruits and may be more acidic depending on the presence of lemon juice in their composition (32). This justifies the lower pH of the Mott’s natural applesauce (US) in comparison to the pH of the other applesauces.

Differences in the pH of the vehicles used for medicine co-administration may affect the dissolution and absorption of drugs. For example, acidic vehicles such as yoghurts, applesauces, jam and honey (pH range 3 to 4.5), have been shown to compromise the chemical stability of acid sensitive drugs, especially in the case of manipulation of enteric coated dosage forms (34).

#### Buffer Capacity

Buffer capacity is higher in yoghurts, applesauces and jam, with lower values measured in formulas and orange squash (Fig. 1b). Significant differences were observed between vehicles of the same subtype (milk and formula (*p* < 0.05)). An accentuated difference was observed between the acid and base buffer capacity of Coca-Cola, whereas this was not observed for the other vehicles. This characteristic of Coca-Cola has been used to temporarily lower the intragastric pH and overcome a clinically relevant decrease of erlotinib bioavailability resultant from concomitant use of the drug with acid-reducing agents, such as proton pump inhibitors (PPIs) (35).

Buffer capacity is especially important to the performance of ionisable compounds since a change in pH can affect the ionisation percentage of these drugs, and thus influence their solubility and dissolution (36). The different results obtained for the buffer capacity of these vehicles suggest that co-administration of a drug with the different vehicles may have an impact on its solubility.

#### Osmolality

The osmolality values of Bramley’s applesauce UK, honey and jam could not be directly measured because they were above the maximum value measurable by the micro-osmometer. Osmolality of these vehicles was obtained by extrapolation of the linear regression of the osmolality of a set of vehicle/water mixtures (% (*w*/*w*)) at various concentrations. Results are presented in Fig. 2.

##### Osmolality of all Tested Vehicles Is Presented in Fig. 1c

Osmolality was generally higher in soft foods than drinks, except for soya yoghurt (298.0 mOsm/kg), Greek yoghurt (393.0 mOsm/kg) and apple juice (693.3 mOsm/kg). The highest osmolality value was observed in honey (6650.0 mOsm/kg) and the lowest in orange squash (22.0 mOsm/kg). The osmolality of the different milk subtypes tested ranged from 200.3 to 318.7 mOsm/kg, which is in accordance with values reported in the literature and likely due to the presence of osmotically active ingredients such as lactose and calcium ions (37). The juices and Coca-Cola were hypertonic with osmolality values higher than 300.0 mOsm/kg (38). Significant differences were observed between vehicles of the same subtype, namely between the different squashes and between the applesauces (*p* < 0.05). Osmolality of the orange squash was 4- and 20-fold lower than osmolality of the blackcurrant Co-Op and Ribena squashes, respectively. These differences can probably be attributed to the higher sugar content of the blackcurrant squashes in comparison to the orange squash (39). Overall, these results are in accordance with previous studies which have shown that osmolality increases with increasing total carbohydrate content, which is strongly influenced by the proportion of monosaccharides, disaccharides or polysaccharides, as well as the levels of organic acids, vitamins and minerals (38). The sugar content, calorific value and osmotic activity of drink vehicles affect the rates of gastric emptying and intestinal absorption (37,40,41). The different osmolality values of the studied vehicles may affect the dissolution behaviour of a drug by inducing changes in the swelling behaviour of the formulation. When the difference in osmotic pressure between the inner and outer part of the formulation decreases, water penetration decreases as well, negatively affecting drug release (42).

#### Surface Tension

Honey and blackcurrant squashes showed the highest surface tension, and soya formula the lowest (Fig. 1d).

The similar surface tension values measured for the dairy vehicles (except soya-derived products) can be related to the composition of these vehicles. Dairy vehicles include surface-active constituents in their composition such as fat, proteins and free fatty acids, which affect the surface tension of these products (43). For the case of soya-derived products, these differ in composition from the other dairy vehicles due to the absence of milk protein and presence of soya protein, which has been shown to lower the surface tension of these products (44). The surface tension of the juices and orange squash is lower compared to the other products due to the higher percentage of water in their composition and to the presence of fatty acids and their salts, which are surface active and reduce surface tension (45).

Differences were observed between the surface tension of Infasoy formula (30.0 mN/m) and the other formulas (40.1 mN/m (Wysoy) and 44.2 mN/m (first milk)), and between the surface tension of the squashes (orange squash: 44.9 mN/m; blackcurrant Ribena and Co-Op squashes: 60.1 and 64.2 mN/m, respectively).

The different surface tension values of the vehicles (including between vehicles of the same subtype) may impact the dissolution rate of a drug by influencing the wetting behaviour of the formulation (46).

#### Viscosity

The viscosity curves of the studied vehicles are shown in Fig. 2. The soft foods studied contain milk and/or macromolecules (*i.e.* starch), which results in a significant increase in viscosity compared to that of the drinks (*p* < 0.05) (47).

The clear differences in viscosity between the drinks and soft foods and between the different soft foods indicate that, depending on the child’s diet, the overall absorption of certain drugs may be altered. For example, in infants, whose diet consists mostly of liquids, the absorption of certain drugs may be increased due to the lower viscosity of the ingested food. Moreover, depending on the volume of vehicle administered, its viscosity can affect the pharmacokinetics of the drug due to alterations of physiological conditions (48). For example, mixing a medicine with a vehicle of higher viscosity such as jam may reduce the diffusion rate of the drug and therefore reduce its overall absorption (39).

### Solubility Studies of Montelukast and Mesalazine

**Fig. 4:**
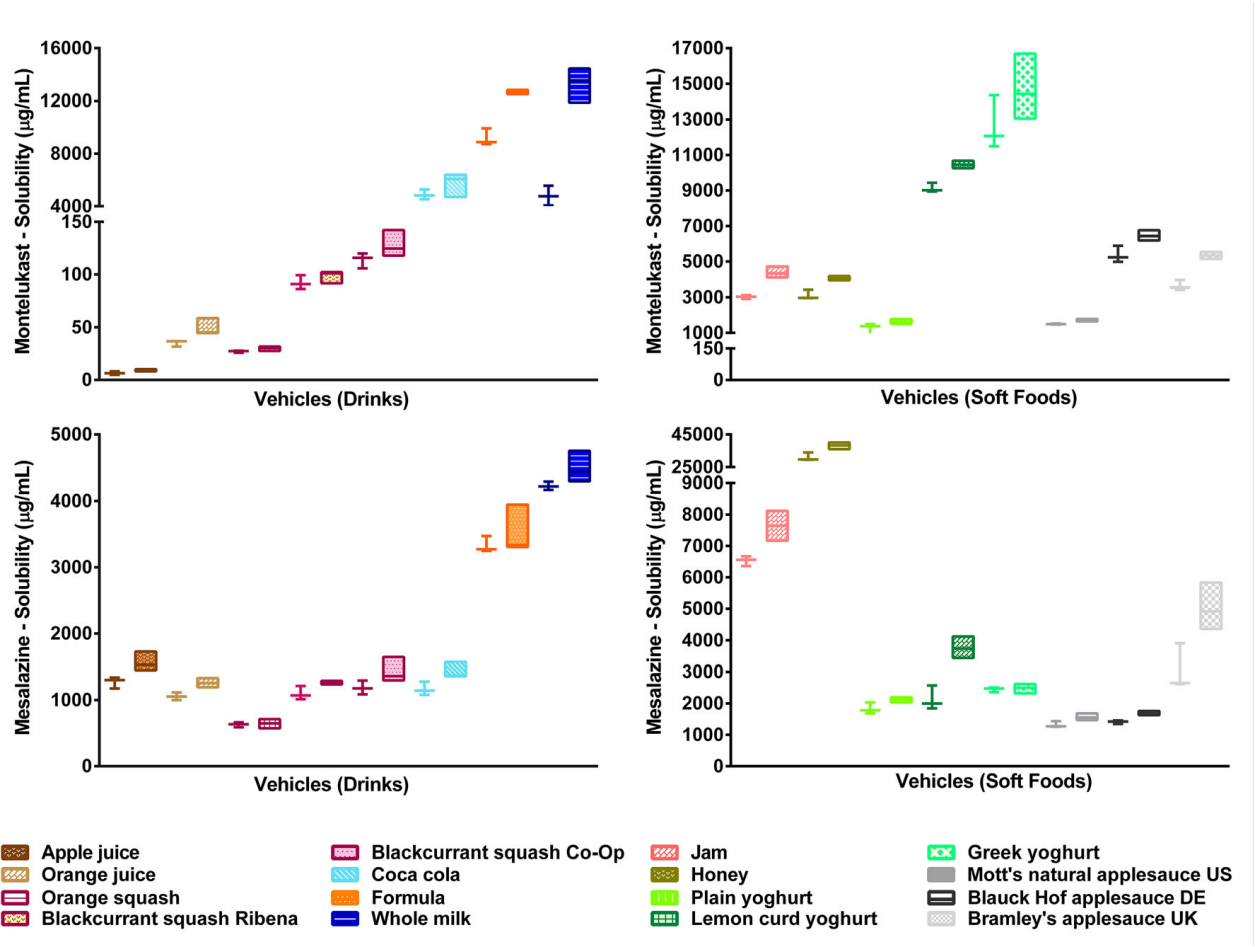
Solubility of montelukast (top section) and mesalazine (bottom section) in the different drink and food vehicles, obtained after 4 h (plot and whiskers) and 24 h (floating bars); values shown represent the 3 replicates measured [each set of colours represents a subtype of vehicles]

Solubility of the two drugs differed in the vehicles studied (Fig. 4).

**Fig. 5:**
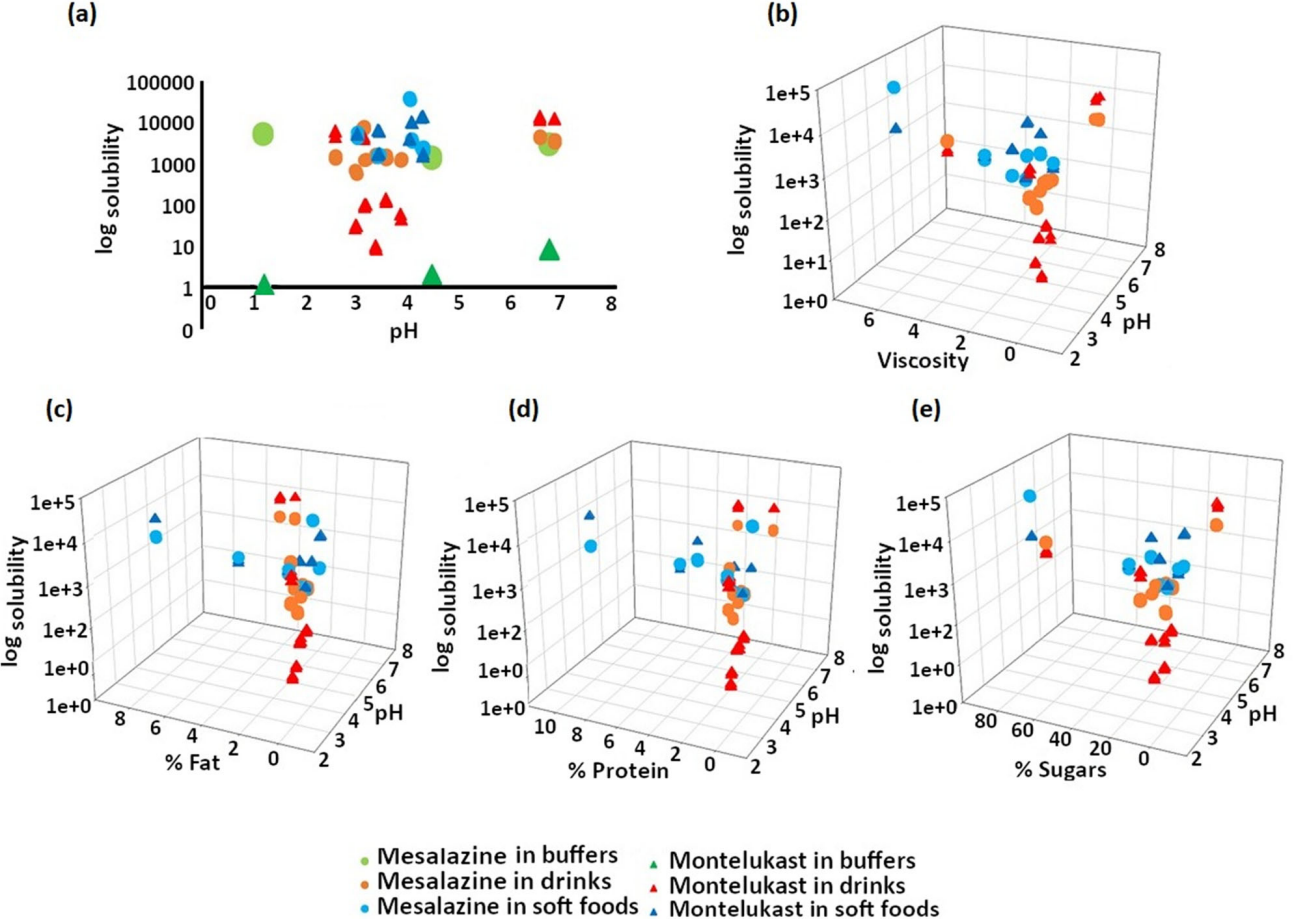
Solubility values (logarithmic scale) of montelukast and mesalazine in the selected media (soft foods, drinks) *vs***a** pH (including solubility in buffers) (2D plot) and *vs***b** pH and viscosity, **c** pH and % of fat, **d** pH and % of protein and **e** pH and % of sugars (3D scatter plots)

Solubility of montelukast in different USP buffers was shown to be pH-dependent (pH 1.2 < pH 4.5 < pH 6.8; Fig. 5a), which is in accordance with previous reports (14). This is attributed to an increased solubilisation at more alkaline pH values, corresponding to the ionisation of the amino group of the compound (pKa 5.8) (15). Solubility of montelukast was generally lower in drinks than in soft foods, except the case of ‘milky’ drinks and Coca-Cola (Fig. 4). In drinks, the lowest drug solubility was observed in apple juice (9 μg/mL; pH 3) and the highest in ‘milky’ drinks (milk and formula: 13.3 mg/mL and 12.7 mg/mL, respectively; pH 6.8), which is likely (in part) due to the pH effect on the solubility of montelukast. In soft foods, the lowest solubility of montelukast was measured in the plain yoghurt (1.6 mg/mL) and the highest in the Greek yoghurt (14.4 mg/mL). Interestingly, the solubility of montelukast in orange squash was 3 and 4-fold lower than in blackcurrant Ribena and Co-Op squashes, respectively, and in Mott’s natural applesauce (US) drug solubility was around 2- to 3-fold lower than in the other applesauces (*p* < 0.05). Differences in drug solubility observed within vehicles of the same subtype and, therefore, same pH range (“pH”), indicate that the solubility of montelukast is also driven by other vehicle physicochemical properties (pH, surface tension, osmolality, viscosity and buffer capacity) and macronutrient composition differences (percentage of sugars, fat and proteins). For example, both sugar content and osmolality values vary within the different applesauces and squashes (“Viscosity”).

For mesalazine, solubility was pH-dependent (solubility in pH 1.2 > solubility in pH 4.5 < solubility in pH 6.8; Fig. 5a). A similar trend has been observed for the solubility of mesalazine in level I and II biorelevant media (49). Lower drug solubility at pH 4.5 could be attributed to the ionisation of this amino acid, which is the lowest at the isoelectric point (pI) of the compound (pH 4.3) and increases as the pH deviates from the pI (50,51). A clear distinction between the solubility of mesalazine in drinks and soft foods could not be made (Fig. 4). Drug solubility was lower in drinks with a pH ~ 4, probably due to the pH effect on drug ionisation and, consequently, solubilisation. Although trends could be seen between solubility and pH for the drinks, differences between drug solubility in drinks and soft foods of the same pH suggested that other vehicle properties, as well as differences in the macronutrient composition of the vehicles, influence drug solubilisation. For example, in yoghurts and applesauces (pH ~ 4), mesalazine exhibited a higher solubility than in drinks of same pH, which could relate to the higher viscosity of these vehicles. For this drug, the highest solubility was obtained in honey (38.4 mg/mL) and the lowest in orange squash (0.63 mg/mL).

Overall, these results demonstrate that mixing these two poorly soluble drugs with soft foods and drinks significantly affects their solubility.

3D correlations of drug solubility values *versus* (*vs*) the vehicle composition (percentage of fat, sugar and protein)/viscosity and pH are presented in Fig. 5b–e.

Analysis of the solubility of montelukast in the different vehicles revealed a crescent shaped trend between the pH and the percentages of fat and protein of the vehicles. The higher solubility of montelukast in the ‘milky’ products (milk, formula and yoghurts) in comparison to its solubility in the other vehicles might be related to the high lipophilicity (clogP 8.79) and high affinity binding of this drug to proteins (16). This is in accordance with drug solubility studies previously conducted in milk which showed a positive relationship between drug lipophilicity, affinity binding to proteins and drug solubility in milk (52).

For mesalazine, a positive interplay was observed for vehicle pH, percentage of sugars and drug solubility in drinks and soft foods. In soft foods, it was possible to observe a positive correlation of drug solubility reliant on an increase of pH and viscosity. A positive correlation between drug solubility and media viscosity has been previously shown for similar compounds, which can justify the higher solubility of mesalazine in soft foods (*e.g.* honey, jam).

#### Statistical Assessment of the Vehicle-Impact on Drug Solubilisation

**Fig. 6:**
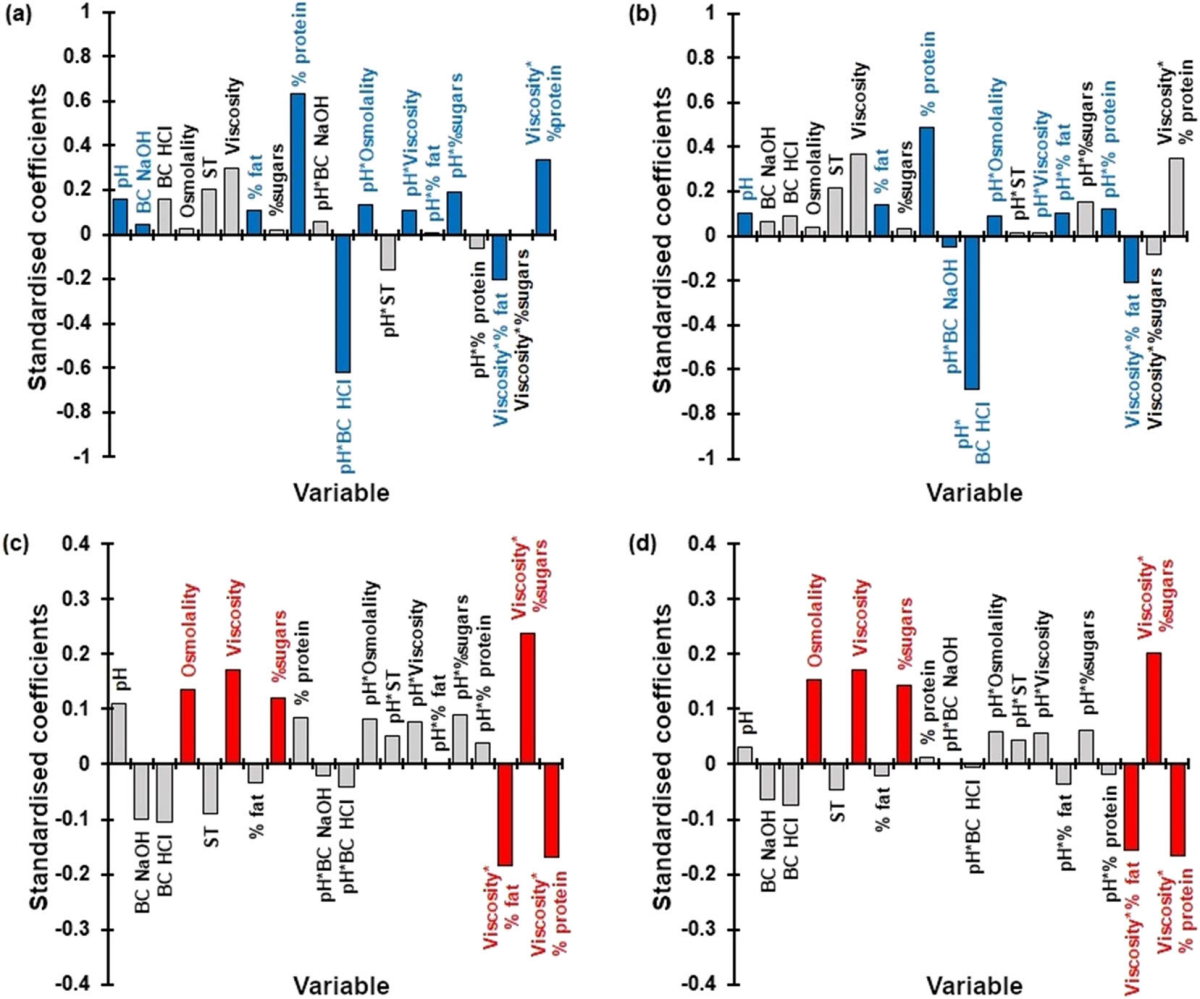
Standardised coefficients corresponding to the variables and interaction studies for the solubility of montelukast (**a**, **b**) and mesalazine (**c**, **d**), at 4 h (left panel; **a**, **c**) and 24 h (right panel; **b**, **d**). Blue and red colours denote coefficients with a significant impact on the solubility of montelukast and mesalazine, respectively (VIP > 1; data not shown). BC = buffer capacity; ST = surface tension

PLS-R analysis was used to understand the vehicle-impact on the solubility of the two drugs. The variables and interactions of the PLS-R models constructed are presented in Fig. 6.

The PLS-R models developed for the solubility of montelukast at 4 and 24 h were defined by 4 and 5 components, respectively, had a good predictive power (*Q*^2^ = 0.66 and 0.78, respectively) and showed a good fit to the experimental values (*R*^2^ = 0.82 and 0.90, respectively). The pH and the percentage of fat and proteins were revealed as the factors with the most significant (positive) impact on the solubility of montelukast, while the buffer capacity of the vehicles had a significant positive impact on the solubility of montelukast at 4 h but not at 24 h (Fig. 6). Significant positive effects of the interaction of pH with osmolality, viscosity and fat, sugar and protein content were observed for this drug, while the interaction of viscosity with protein content was shown to have a significant positive impact at 4 h but not at 24 h. The interaction of pH with buffer capacity and viscosity with fat content were shown to negatively impact the solubility of montelukast.

For mesalazine, the PLS-R models constructed for drug solubility at 4 and 24 h showed a good fit to the experimental values (*R*^2^ = 0.98 and 0.94, respectively), a good prediction power (*Q*^2^ = 0.95 and 0.91, respectively) and were defined by 3 and 2 components, respectively. Vehicle viscosity, osmolality and sugar content were the significant factors impacting the solubility of mesalazine (all positive effect), while significant effects from the interactions of viscosity with protein and fat content (negative) and the interaction of viscosity and sugar content (positive) were revealed (Fig. 6).

The difference in the vehicle variables (physicochemical properties and/or macronutrient composition) that impact the solubility of each drug suggest that the effect of the co-administered vehicle also depends on the properties of the drug (namely, lipophilicity, pKa, acid/base properties). Knowledge of the physicochemical properties and macronutrient composition of the vehicles and drug/formulation physiochemistry could help predict the potential vehicle-impact on drug solubility and should be considered during compatibility assessments of the vehicle-drug product. For example, for drugs like montelukast, solubilisation may be increased when the formulation is mixed with a dairy vehicle than when mixed with juice. Moreover, the different results obtained for each drug highlight the importance of considering the nature of the vehicle utilised in common practice and possible effects of a change in recommendation. This is of particular importance considering that even though the recommendations for the administration of Singulair® granules (montelukast formulation) are to mix with ‘a spoonful of cold soft foods’, differences in drug solubility were observed for soft foods of the same subtype (*e.g.* between plain and Greek yoghurts), demonstrating the potential risks of this practice. Moreover, the recommended vehicle to mix with Pentasa® granules (mesalazine formulation) is orange juice; however, if the juice is substituted for another vehicle such as formula, due to the child’s diet/age, the medicine co-administration practice may result in a different drug solubilisation and, consequently, *in vivo* drug performance.

Ultimately, medicine co-administration with different vehicles may alter the clinical performance of a drug by affecting not only its solubility but also dissolution performance and, consequently, bioavailability. Although in some cases this can be beneficial, the risk of reduced efficacy and increased toxicity associated with this medicine administration practice is concerning.

## CONCLUSION

(Soft) foods and drinks are commonly used to facilitate medicine administration to the paediatric population in order to improve palatability and enhance compliance. In this study, 26 vehicles that are commonly mixed with oral medications for paediatric administration were characterised in terms of their physicochemical properties and macronutrient composition. Differences were observed across the range of food and drinks, notably not only among vehicles, but also within vehicles of the same subtype These differences are expected to affect drug behaviour, such as its solubility and dissolution, especially in the case of a poorly soluble drug. Solubility studies of two model compounds, performed in selected drinks and soft foods resulted in considerably different solubility values in each vehicle. The solubility of the drugs was significantly affected by the vehicle physicochemical properties and characteristics, with the solubility of montelukast driven by pH, fat and protein content and the solubility of mesalazine by vehicle viscosity, osmolality and sugar content. This vehicle-dependent impact on drug solubility could compromise drug bioavailability and should be taken into consideration during paediatric product development.
